# Stakeholder perspectives on mental health systems and services for emerging adults in Barbados

**DOI:** 10.3389/fpubh.2026.1779099

**Published:** 2026-06-03

**Authors:** Amanda Kellman, Heather Harewood

**Affiliations:** Department of Preclinical and Health Sciences, Faculty of Medical Sciences, The University of the West Indies, Bridgetown, Barbados

**Keywords:** emerging adulthood, help-seeking, mental health services, mental health systems, stigma, user perspectives

## Abstract

**Introduction:**

Emerging adulthood (ages 18–29) is a distinct developmental stage marked by significant psychological, social, and biological transitions, including identity formation, growing independence, and shifts in educational, employment, and relational roles. These increasing responsibilities and societal expectations heighten vulnerability to the onset or worsening of mental health challenges such as anxiety, depression, and substance use. Therefore, emerging adulthood is a critical period for mental health intervention. In Barbados, the post-pandemic period has seen a sharp escalation in mental health needs, particularly among young people, evidenced by a 100% rise in individuals seeking mental health services, increased demand for Community Mental Health services, and higher suicide rates alongside a surge in pandemic-exacerbated mental health cases. This study aimed to explore mental health systems and services for emerging adults in Barbados from the perspectives of local stakeholders (clients and providers of public, private, and NGO-based mental health services).

**Methods:**

This qualitative exploratory study, grounded in an interpretivist paradigm, used purposive sampling guided by saturation. A semi-structured interview guide explored the interplay between individuals’ mental health needs and the services provided. Seventeen interviews (30–45 min) were conducted with stakeholders recruited through gatekeepers in the governmental and non-governmental mental healthcare sectors. Interviews were conducted in person or via encrypted video conferencing, transcribed in a modified verbatim format, and analyzed using Hamilton’s five-step Rapid Qualitative Analysis.

**Results:**

Four preliminary themes emerged from the data reduction process: “Perspectives on Mental Health and Mental Illness,” “Framing Emerging Adulthood,” “Health Service Needs,” and “Facilitators and Barriers to Help-Seeking.” These themes explicate how stakeholders conceptualize mental health and service use factors.

**Discussion:**

These insights highlight the pressing importance of implementing culturally relevant, developmentally appropriate mental health reforms that support emerging adults and strengthen their long-term resilience.

## 1 Introduction

Emerging adulthood is a distinct development stage (approximately 18–29 years old) characterized by profound psychological, social, and biological transitions (1, 2). During this stage, individuals typically navigate increased independence, identity formation, educational and employment pursuits, and shifts in family and peer relationships (1). There are increasing responsibilities and societal expectations during emerging adulthood, and these transitions increase the vulnerability of emerging adults to new onset of mental health challenges, including anxiety, depression, and substance use, and to the exacerbation of pre-existing mental health disorders (3).

The landscape of global health is increasingly acknowledging the profound impact of mental health challenges, particularly among young populations. In 2019, out of 2,516 million individuals worldwide aged 5 to 24 years, 293 million (11.6%) were living with a diagnosable mental disorder (4), the leading cause (20.3%) of years lived with disability (YLDs) from all causes in this age group (4). Globally, it is estimated that 14.3% of adolescents suffer from mental ill health, with onset peaking around age 14 years (3–5) and persisting into emerging adulthood (age 18–29 years) (1). Mental disorder prevalence was twice as high among 15- to 19-year-olds (14.0%) and 20- to 24-year-olds (13.6%), respectively, compared to 5- to 9-year-olds (6.6%) (4), highlighting the vulnerability of transitional developmental periods, including emerging adulthood, to mental disorders.

The COVID-19 pandemic has contributed to the increased prevalence of mental health disorders among adolescents and young adults (6). Studies in the USA and Portugal showed that the 18–29 year age group was disproportionately affected by anxiety and depression levels during the pandemic (7, 8) and experienced impairment of their wellbeing (8). Emerging adults were thought to be especially vulnerable to the mental health effects of COVID-19 because of the disruption to their developmental processes, including autonomy and achievement of financial responsibility (7). Background vulnerability, which can be exacerbated by contemporary stressors, underscores the need for comprehensive health care for emerging adults (8).

There is a high burden of youth mental disorders in the Caribbean, whereby 20 to 25% of anglophone adolescents experience mental health problems (9), depression rates among adolescents were found to be 15% higher than the global average, and one in four adults has a diagnosable mental health condition (10). The Caribbean Public Health Agency (CARPHA) reported that anxiety disorders and depression accounted for 50% of mental health conditions, and suicide rates showed upward trends, particularly among Caribbean youth (10). Specifically in Barbados, within both the 20–24 and 25–29 age groups, anxiety (4.1 and 4.6%, respectively) and depressive disorders (3.9 and 4.4%, respectively) were among the top 10 causes of disability adjusted life years (DALYs) in 2019 (11). In Barbados, in the post-pandemic period, there has been a 100% increase in individuals seeking psychological services (12). In 2023, attendance and demand for Community Mental Health services at the polyclinics increased (13). Additionally, the Ministry of Health and Wellness has reported increased suicide rates in 2022/2023 and a surge in mental health cases exacerbated by the impact of the COVID-19 pandemic (14). This highlights the substantial public health burden and long-term implications for health systems in Caribbean Small Island Developing States (SIDS).

The Socioecological Model (SEM; based on Bronfenbrenner’s Ecological Systems Theory) provides a useful framework for understanding how societal risk factors influence mental health and wellbeing during emerging adulthood. The five-level model proposes that health outcomes are shaped by dynamic interactions across multiple levels of influence, including the individual, interpersonal, community, organizational, and societal or policy levels (15, 16). At the individual level, emerging adults may experience risk factors such as identity instability, financial insecurity, substance use, and difficulties coping with developmental transitions (17). At the interpersonal level, strained family relationships, peer conflict, intimate partner difficulties, and limited social support may contribute to emotional distress and poorer mental health outcomes (18). Community and organizational factors include unemployment, educational stress, community violence, housing instability, stigma surrounding mental illness, and limited access to developmentally appropriate mental health services, all of which may hinder help-seeking and wellbeing among emerging adults (19). At the broader societal level, structural inequalities, poverty, discrimination, gender norms, and inadequate mental health policies inherited during colonialism created conditions that militated against mental wellbeing. The British colonial period and the associated displacement and forced labor of slavery were a period of increased vulnerability for mental ill health. The punitive response to expressed mental ill health further perpetuated disparities in care delivery (13, 14, 20). Collectively, the levels of the SEM highlight that mental health challenges during emerging adulthood are not solely individual problems, but are shaped by interconnected social, structural, and environmental conditions that require multilevel interventions and systemic responses. The SEM lens provided the framework for the conduct of this study.

It is imperative for health systems to address the sub-optimal mental health diagnostic and treatment pathways that pave the way for poorer physical, mental and life course outcomes as adolescents transition into adulthood (4, 5). Mental health systems are the comprehensive frameworks of policies, infrastructure, funding, and human resources, ideally designed to organize and deliver tailored, responsive and accessible interventions, treatments, and prevention programs (21). Barbados provides public ambulatory (polyclinic) and tertiary (hospital-based) mental health services free at the point of delivery as part of a universal health care package (22). There is currently a service gap as there are no dedicated emerging adult mental health services. Young people attend pediatric mental health services like the Child and Adolescent Mental Health Clinic (CAMHC) from ages 3 to 17 years until transfer to the “adult” clinic at age 18 years (22). Public provision is complemented by ‘fee for services’ private and NGO providers who also provide mental health care. Like many SIDS, distinct challenges such as limited human resources and budgets (fiscal constraints) are recognized health system constraints (23).

During the time of this study there was ongoing national consultation on mental health reform in Barbados (24). Within this context, recent increases in suicides and other mental health disorders, particularly among young adults in Barbados, have led the government to institute several mental health-promoting initiatives, including a 24-h mental health hotline, Lifeline Barbados (25). The increased prevalence of youth mental health disorders, societal concern, and political will to engage experts and society in the national consultation presented a unique opportunity to systematically explore the perspectives of key stakeholders and generate findings that could contribute to the ongoing local discussions and reform processes.

This study explored stakeholders’ (emerging adult service users and providers within public, private sector and NGO-based settings) perspectives on the current mental health systems and services for emerging adults in Barbados. Three objectives guided the study: (1) to explore the demand/need for services for emerging adults in Barbados, (2) to identify barriers and facilitators to access and utilization of mental health services for emerging adults in Barbados, and (3) to gather emerging adults’ perspectives regarding the ability of mental health services to meet their needs.

This study contributes to a more nuanced understanding of the mental health landscape for emerging adults in Barbados, providing evidence-based insights to potentially inform policy development to enhance design and delivery of services for emerging adults in Barbados. Findings may be transferable to other Caribbean SIDS.

## 2 Materials and methods

### 2.1 Study design

This research is framed within an interpretivist philosophy, focusing on the subjective meanings and lived experiences that individuals attribute to their social world (26). In alignment with this worldview, the study adopted an inductive approach, in which data collection preceded theory formulation, allowing themes to emerge organically from participants’ narratives (27). The central research strategy is phenomenology, specifically chosen to explore and describe the “essence” of human experience as it relates to the phenomenon under investigation (28, 29). Finally, the techniques and procedures involved semi-structured interviews (SSIs) to enable open-ended dialogue, followed by rapid qualitative analysis (RQA) to synthesize and interpret the resulting transcripts (30). Phenomenology informed the data collection, given the objective of deeply exploring and capturing the lived experiences of this understudied and vulnerable group. As the study took place during ongoing mental health reform in the country, a pragmatic decision was made to conduct rapid qualitative analysis with inductive theme development. RQA was chosen to quickly but rigorously generate information relevant to the dynamic interplay of emerging youth mental health needs and the national consultation occurring in ‘real-time’ on the island. This combined approach enabled an efficient analysis that could inform policy and practice without losing deep perspectives on how individuals make sense of their world (31, 32). The Consolidated Criteria for Reporting Qualitative Research (COREQ) were followed to enhance the study’s overall rigour (33).

### 2.2 Setting

This study took place in Barbados, an Eastern Caribbean high-income SIDS with an estimated 2024 population of 282,467, of which approximately 13% of the population (36,694) was between the ages of 20 and 29 (34, 35). Both public and private agencies provide health care, including mental health care (22). In the public sector, mental health care is delivered at the Psychiatric Hospital, Queen Elizabeth Hospital, and nine polyclinics (22). Providers include psychiatrists, psychologists, community mental health nurses, and trained counsellors.

### 2.3 Target population, sampling, and recruitment of participants

The target population of this study consisted of key stakeholders, namely, emerging adults with the lived experience of using or attempting to access mental health services within the public or private sector, and mental health professionals involved in the treatment of emerging adults for more than 6 months (Table 1). Stakeholders were eligible to participate if they were aged 18 years or older, resided or worked in Barbados, and had direct experience with emerging adult mental health needs and service delivery.

**Table 1:** Socio-demographic characteristics of the stakeholders.

Participant no.	Age	Gender	Stakeholder group	Educational attainment	Employment status	Mental health care accessed
P1	25	F	Emerging adults	Bachelor’s degree	Unemployed	Public/private
P2	26	F	Emerging adults	Secondary school	Employed full-time	Public/private
P3	29	M	Emerging adults	Bachelor’s degree	Employed full-time	Private
P4	18	F	Emerging adults	6th Form	Full-time student	Public/private
P5	21	F	Emerging adults	Secondary school	Unemployed	Public
P6	29	F	Emerging adults	Bachelor’s degree	Unemployed	Public
P7^a^	21	F	Emerging adults	6th Form	Full-time student	Private
P8	19	F	Emerging adults	Secondary school	Employed full-time	Public
P9	25	F	Emerging adults	Secondary school	Full-time student	Public
P10	24	F	Emerging adults	6th Form	Full-time student, full-time employed	Private
P11	25	F	Emerging adults	Secondary school	Employed full-time	Public
P12	26	F	Emerging adults	Bachelor’s degree	Employed full-time	Public
P13	29	F	Emerging adults	Master’s degree	Employed full-time	Public/private
P14	–	F	Mental health professionals	–	–	Private
P15	–	M	Mental health professionals	–	–	NGO
P16	–	F	Mental health professionals	–	–	Public/private/NGO
P17^a^	18	F	Emerging adults	Secondary school	Full-time student	Public/private

a Participants were referred to the study through tertiary counselling centers.

A purposive sampling strategy was used (36). Thus, participants were selected because of their perceived value in helping the researcher to explain the topic. Maximum variation was employed to achieve a heterogeneous sample and thus enhance the likelihood of capturing the widest range of perspectives. Categories included age, gender, socioeconomic background, geographic location, and type of stakeholder role (37). Snowball sampling was employed to identify additional key informants, particularly those from hard-to-reach or marginalized subgroups (37).

Following IRB approval, formal permission was requested from governmental and non-governmental organizations providing mental health services to access the research sites. Gatekeepers, who were in management or supervisory positions, provided physical and social access to potential participants. Information about the research study and the principal investigator (PI), AK (a female registered clinical psychologist and master’s level student trained in qualitative methods), was provided to the gatekeepers, along with recruitment materials. Gatekeepers were asked to share recruitment materials with potential research participants who satisfied the study’s inclusion criteria. Potential research participants were asked to contact AK directly, and AK made the final selection after reviewing participants against the study’s inclusion criteria. Gatekeeper-assisted recruitment was augmented with the use of WhatsApp posts and flyers.

### 2.4 Methods of measurement/assessment/quality assurance

This study followed a qualitative design, ensuring a deep focus on rich, non-numerical data supported by techniques to maintain methodological rigor and authenticity. For this study, in-depth SSIs were used to explore the participants’ lived experiences. An SSI guide (Table 2) was developed by AK and HH (a female researcher with doctoral training in qualitative methods serving as AK’s research supervisor). The guide was informed by a previously conducted (AK and HH) scoping review and was aligned with a SEM framework (16, 38). Questions were structured around the definition of mental health; participants’ subjective wellbeing and mental health experiences; perceptions of mental health needs among emerging adults; experiences with service access or delivery; perceived barriers and facilitators to care; mental health policy; recommendations for improving system responsiveness; and emerging adulthood. Methodological rigor was assured through the use of contact summary sheets, field notes, debriefing, and reflexivity.

**Table 2:** Interview guide (shown without prompts).

No.	Question
1	What does mental health mean to you?
2	How good or bad is your mental health today?
3	What is your personal experience with mental illness?
4	What initially prompted you to seek treatment for your mental health?
5	How long have you been seeing a psychiatrist, psychologist, or other mental health professional?
6	What has been your experience in seeking help for your mental health concerns?
7	What is your opinion on mental health services for young people?
8	What makes it easy for young people to seek support for mental health concerns?
9	What are the challenges that young people face in seeking support for mental health concerns?
10	What other means of support may young people be assessing?
11	What are the main needs of young people in terms of services?
12	What should services look like or encompass for emerging adults?
13	How likely are you to recommend these mental health services to a family member or friend around your age?
14	Since the start of the COVID-19 pandemic, there has been an increase in the number of young people experiencing mental health concerns and expressing a need for mental health services. What do you make of this?
15	How does climate change impact on mental health?
16	Can you tell me what you know about mental health policies in Barbados?
17	What do you understand by the term ‘emerging adulthood?’
18	How do you view this phase of development impacting your mental health?
19	How might this phase of development impact on the mental health of other young people?

### 2.5 Data collection, management, analysis, and considerations of sample size

The SSI was piloted on June 13, 2024, and after discussion and revisions, data collection continued until May 16, 2025. Data collection followed standardized procedures for administering the SSIs to ensure consistency and quality. Participants were recruited on-site in the psychiatric hospital, community clinics, university or college counselling services and lecture rooms, private practices, and youth-focused NGOs. Those expressing interest were provided with a participant information sheet detailing the study’s purpose, procedures, risks and benefits, confidentiality protections, costs and payments, and the voluntary nature of participation. There were no incentives for participation. Consent (written or verbal depending on the modality of the interview) was obtained prior to participation.

Semi-structured interviews were conducted either face-to-face or via a secure online platform (e.g., Zoom), depending on the participant’s preference and time constraints/availability. Each interview lasted 30–45 min. A pilot interview was conducted by AK, then reviewed collaboratively with HH. HH offered feedback on establishing rapport, suitable prompts, etc. and AK completed the remaining interviews, which were audio recorded with participant permission and transcribed in a modified verbatim format by AK first using Cockatoo for artificial intelligence (AI) (57) and then verifying the transcript against the audio using MS Word and a media player. HH checked 25% of transcripts against audio recordings for accuracy. Standard English substitutions were used for Bajan dialect (local “broken” English) and were included in square brackets. Transcripts were anonymized prior to analysis and were not returned to participants for comment and/or correction. Field notes made during the interviews captured nonverbal cues, contextual reflections, and preliminary analytic impressions.

Audio recordings were promptly downloaded into an electronic format on an external hard drive within 24–48 h. Audio recordings were permanently deleted from the recording device immediately after confirming the accuracy and completeness of transcriptions. Each interview was identified by an alphanumeric code including the type of institution, date, and interview number. Electronic data was securely encrypted with a password using 7-Zip and shared between AK and HH using a private MS SharePoint Online folder.

Transcripts were read repeatedly to ensure familiarization with the data. RQA was conducted using Hamilton’s five-step framework, which involves (1) creating neutral domain names for each question, (2) creating a template to use for summarizing data using the neutral domains (Table 3), (3) testing the template for usability with each interview and relevance to project goals, (4) transferring summaries to a matrix with the domains listed across columns and the participants listed across rows, and (5) synthesizing the data within cases and across the dataset (30). The template was validated (*n* = 4) by AK and HH in an independent, iterative, collaborative process. First, the researchers completed the templates separately, then discussed the data that had been summarized for each of the four cases/participants. Rapid techniques are used to reduce time and costs, increase the amount of data collected, improve efficiency and accuracy, and obtain a closer approximation to the narrated realities of research participants (39). The overarching goal of using rapid qualitative methods is to generate actionable results that may impact practice (40). Barbados is in the process of revising its Mental Health Act to make it more rights-based (24). In this case, RQA was used because the Barbados Mental Health Commission was in the process of sensitizing the public to the draft legislation, and this approach would facilitate a time-sensitive contribution to those national discussions.

**Table 3:** Interview summary template.

Emerging adult mental health services interview summary template		
Prepared by:		
Interviewer:		
Interview date:		
Respondent demographics:		
No.	Domain	Notes/important quotations
1	Understanding of mental health	
2	Subjective wellbeing	
3	Personal experience	
4	Help-seeking timelines	
5	Length of time/frequency of treatment	
6	Benefits of services	
7	Weaknesses of services	
8	Help-seeking experience	
9	Mental health services for young people	
10	Facilitators to help-seeking	
11	Barriers to help-seeking	
12	Informal support	
13	Needs of young people	
14	COVID-19 increases in service utilization	
15	Navigation of services in the COVID-19 pandemic	
16	Perceived climate change impacts	
17	Mental health policies	
18	Emerging adulthood	
19	Vulnerabilities in emerging adulthood	
20	Other observations	

Sample size was determined iteratively through ongoing data collection and analysis, and was guided by the principle of saturation (41). Saturation was defined as the point at which no new themes emerged (41). In this study saturation on several themes was observed by the 17th participant, which is in keeping with the literature that states that saturation occurs between 9 and 17 interviews (42). A pragmatic decision was made to complete the data collection at this point in view of the goals of conducting a rapid analysis in the context of the ongoing local mental health consultation.

### 2.6 Ethical considerations

Ethical approval was obtained from The UWI Cave Hill Research Ethics Committee and from the participating health institutions prior to study commencement, and the study was conducted in accordance with the principles of the Declaration of Helsinki (43). All participants provided informed consent and were assured of their right to withdraw at any stage without consequence. Confidentiality was maintained by replacing identifying information with pseudonyms and storing all data on encrypted, password-protected devices accessible only to the research team.

Given the sensitivity of discussing mental health experiences, participants were provided with a coping skills card and a list of local support resources and offered referrals. Since the researcher is a mental health provider, special care was taken to minimize power differentials, particularly when engaging emerging adults, unemployed individuals, or persons with histories of service disenfranchisement.

### 2.7 Reflexivity statement

The primary researcher, AK, is a registered clinical psychologist working in private practice. She approached this study with an awareness that her professional training, clinical experiences, and personal assumptions could influence the research process. As a result, AK used several reflexivity techniques to ensure methodological rigor and limit the influence of her own beliefs, judgments, and practices on the research being conducted (44). These techniques included keeping a reflexive journal, peer debriefing, and critical self-reflection (45). First, reflexive journaling was used continuously to document thoughts, feelings, observations, and reflections. Immediately after each interview, AK recorded observations regarding participant interactions, decisions about data collection and analysis, and any assumptions or biases in the questions or reactions. This process allowed AK to critically examine how her professional background, positioning as a clinician and researcher, and prior experiences, may have shaped her interactions. Next, regular peer debriefing was conducted with HH to discuss field notes, contact summary sheets, and the data collection and analysis processes. HH offered feedback, guidance, and alternative views where appropriate. Lastly, AK engaged in critical self-reflection to examine how her dual roles, values, and commitments to culturally responsive practice influenced interactions with participants and the framing of results. Such reflection ensured that her interpretations remained grounded in participants’ lived experiences rather than solely in professional or theoretical perspectives. Together, these reflexive strategies supported a transparent and ethically attuned research process, reinforcing the study’s commitment to rigor, authenticity, and cultural responsiveness.

## 3 Results

Seventeen stakeholders (15 women, two men; P1–P17) participated in the study, drawn from the two key groups, emerging adults (*n* = 14), aged 18–29 (M = 23.9; SD = 3.93), and mental health professionals (*n* = 3). All emerging adult participants identified as Black Barbadians and lived in households with other people. One participant (service user) dropped out of the study, citing personal/time conflicts. Four preliminary themes (PT1–PT4) emerged from the data reduction process: “Perspectives on Mental Health and Mental Illness,” “Framing Emerging Adulthood,” “Health Service Needs,” and “Facilitators and Barriers to Help-Seeking.” These themes will form the backdrop for presenting the results.

### 3.1 Perspectives on mental health and mental illness (PT1)

Participants described mental health as overall emotional, psychological, and social wellbeing, thus reflecting a holistic, non-disease-based perspective.

“*Emotional, psychological well-being. So like emotions referring to the feelings and in psychological, more so like cognitive functioning, thought processes, stuff like that.”* (Participant 12, female service user).

“*Being in a place where what you think, how you feel, how you behave, all of those things are in one accord and you are able to function. You’re able to engage in activities. You’re able to interact with other persons.*” (Participant 14, female service provider).

Mental health was seen as an important attribute, and emerging adults, in particular, emphasized its personal significance.

“*Mental health is something that was very important to me. I used to… learn how to manage the stress and a lot of things like that.*” (Participant 4, female service user).

In contrast, mental illness was defined by participants as a diagnosable condition like anxiety or depression, causing significant distress or impairment in daily life.

“*Mental illness [illnesses] are the actual… diagnosis [diagnoses] that you can get… Mental illness comes once there’s been an identified problem with your mental health so if they notice that you are unstable, you are really moody… then they might diagnose you with a mental illness.*” (Participant 8, female service user).

“*Mental illness would be something like anxiety or depression.*” (Participant 4, female service user).

Furthermore, mental illness was seen as multifaceted with multiple causes.

“*You will not end up developing it like out ah [of] de [the] blue right? It may be something dat [that]is within you from your genetics or whaever [whatever] de [the] case might be or something dat [that is] traumatic could happen to you. And it’s something dat [that] not necessarily would be able to be fixed.*” (Participant 3, male service user).

“*You would have to go through certain things to develop the mental illness. Like traumatic events. Lifestyle changes. Moving to a new place.*” (Participant 17, female service user).

Yet, mental illness was also often seen as hidden, making it difficult to identify or address early.

“*The depression used to affect me from every [ever] since. But… I never knew that that’s what it was.*” (Participant 3, male service user).

“*You really cannot tell by looking…there are like certain behaviors and stuff that ya [you] need to have but like to just look at somebody and say, yeah that body got [has] a mental illness, I think that’s not necessarily something I could do.*” (Participant 1, female service user).

This apparently dormant nature of deteriorating mental ill health sometimes developed into a precipitous clinical presentation requiring acute/emergency care, as outlined by one participant.

“*I never wanted to be in here. My mom put me in. She heard from a friend whose child has a similar problem, and yeah, she was worried obviously.*” (Participant 4, female service user).

Participants’ identification of the complexity of mental health and illness was echoed in their descriptions of the multi-faceted nature of emerging adulthood.

### 3.2 Framing emerging adulthood (PT2)

According to participants, emerging adulthood is a challenging, transitional time marked by autonomy and achievement.

“*I think it begins when you feel like you have to do everything on your own. […] Having to survive, you would say*. *Due to the economy and stuff like that, that’s been happening…*” (Participant 2, female service user).

“*Someone who is stepping out from being a teenager who’s being more independent, making more, indeed, choices on their own. And that person who has to be the sole person dealing with those choices that they make.*” (Participant 14, female service provider).

This may generate challenges with defining societal roles and identity formation, and most prominently, increasing financial responsibility.

“*Responsibilities… come in a lot of different forms… children… bills… taking care of your family, whether it’s your mother or your father, or… taking care of yourself*.” (Participant 3, male service user).

“*This is… where persons are now stepping into adulthood…transitioning from being a teenager into being an adult in society… from the end of the adolescent or teenage stage to late 20s and there is a shift in responsibilities… your main focus is getting out of secondary school, getting out of community college. But after… you are transitioning to being an adult the focus shifts to getting a job, maybe furthering your studies at university, building a career, probably thinking about marriage or starting a family… So, for emerging adults I think that there is sense of instability because trying to navigate these new responsibilities…now being somewhat independent you tend to struggle a little bit… some persons now have to pitch in to the family, and trying to figure out who they are as an adult because they are somebody else as a child in secondary school… but now you are seen as “grown” in the eyes of many and there is a struggle for self-identity, like, ‘Who am I as a [an] adult? Who am I going to be during adulthood?’ So I think that these responsibilities, these struggles, create some form of like instability in a way and vulnerability to mental health issues….”* (Participant 9, female service user).

*“For me, I think being an adult is somebody who can make decisions and handle the consequences of their decisions. And has the ability to make decisions. So you have the money for it, you have the support for it, you know?”* (Participant 12, female service user).

Participants articulated the potential disruptors of mental wellbeing such as financial responsibility. They also shared expectations about what the ethos and components of services for emerging adults should ‘look like’.

### 3.3 Health service needs (PT3)

Emerging adults expressed a desire to be seen as normal and to have the right to self-expression.

*“…she makes you feel comfortable so… it does not feel like you are in a room and she’s just sitting down like taking notes, she just talk [talks] to you like a normal…*” (Participant 2, female service user).

*“Having the opportunity to actually be heard, because that was another thing that I did not have a lot of growing up… having somebody to talk to about my problems and things of that nature.”* (Participant 3, male service user).

In contrast to these positive experiences, some people described dissimilar health services encounters, characterized by a feeling of disconnection from their providers.

“*People can seem like they do not care.*” (Participant 12, female service user).

“*And like, talking to her, seeking help at that point in time, I realized that she was almost distant… [W]hen I was talking about certain things… she did not [wasn’t] really present wid [with] wha [what] I was talking about. So I started to say, “You know what, I do not think I can go through with the service anymore.*”” (Participant 3, male service user).

“*I think that the connection is what is missing. So they would complain about how de [the] psychiatrists treat them… How de [the] psychologists treat them.*” (Participant 16, female service provider).

Participants appeared to be well informed about the services available locally, based on their ability to list specific services. However, they highlighted that services may not be youth-specific, may be hard to access, and that some providers lack awareness and engagement strategies for emerging adults.

“*I do not think it’s really a difference between if you are young or old.*” (Participant 5, female service user).

“*I guess because at the [named healthcare institution] they already have a lot of patients to deal with and stuff so it’s like they are already swamped with so many things that are going on it’s like you are not getting the full attention so that they can listen out to you.*” (Participant 2, female service user).

“*I was gonna say, at one point in time I thought it was that I needed younger people. But I realized it could be the oldest or the youngest person. You could bring a baby. But it still really will not do a thing. I think it just needs more people who actually like their job and not just doing the job to get money.*” (Participant 5, female service user).

Emerging adults identified an unmet need for school-based early intervention in Health and Family Life Education (HFLE) or more guidance counsellors in secondary schools. Other services such as therapy, counselling, assessments, support groups, hotlines, mentoring, community outreach programs, and access to better medications were seen as essential.

“*We do not have patient-informed care so we do not listen to patients when they talk about what they think they need from their caregiver. So, I think that’s something I would like as well. HFLE in schools.*” (Participant 1, female service user).

“*I find that it’s not much going on, especially in primary, well not primary, secondary, where a lot of students are just lashing out at others and not [do not] really have someone to talk with. For example, the guidance counsellor would be nice.*” (Participant 2, female service user).

“*I use the hotline sometimes… It has actually saved me twice.*” (Participant 5, female service user).

Participants shared the need for such services to be accessible, affordable, and cost-effective. They noted that public services lacked human and financial resources to provide adequate services for emerging adults and that private services were too costly.

“*I do not know if there are much [many] resources publicly because I know a lot of people might cannot afford private but I do not think it’s like enough people out there public-wise to help young people in terms of mental health problems and stuff like that. Because I just see a lot going on on social media where people are seeking out for help but they have to end up going private but they do not have the money to go private so I guess there are not much resources public-wise.*” (Participant 2, female service user).

Participants further explained that the ability to actualize needs and preferences for mental health care models was modulated by factors influencing access to services.

### 3.4 Facilitators and barriers to help-seeking (PT4)

Help-seeking behaviors were influenced by both enabling and inhibiting factors. Access to information, particularly from peers, the social media presence of the Psychiatric Hospital, knowledge of services, and psychosocial support encourage help-seeking among emerging adults.

“*I’ve had people call me and ask me about who I’ve seen or if I could make a referral or what the process here [is] because I’ve been kinda [kind of] open coming to Psychi [hospital] and taking medication… if we could demystify it and not make it seem like a big scary thing, that would be great.*” (Participant 12, female service user).

“*Psychiatric Hospital, I think, has an Instagram and Facebook page and they are trying to be more active on it.*” (Participant 1, female service user).

“*I think if they have support from the people around them, it’ll make it easier.*” (Participant 4, female service user).

“*Easy access… having an idea that it’s there, having a little overview of who are the persons dealing with it, and having the opportunity to come into the space.*” (Participant 14, female service provider).

Stigma, particularly around the Psychiatric Hospital, a lack of psychosocial support, direct and indirect costs, and misconceptions discourage help-seeking among emerging adults. In some cases, stigma emerged from the participants’ family members.

“*…my mother wants to change me to go to a private psychiatrist. She does not like the thought of me being at the Psychiatric Hospital… She keeps saying that she does not think I’m ill or … she sees the hospital as a place for… crazy or mad people… I did not like the comment. … She does not like me being there*.” (Participant 17, female service user).

“*Only crazy people does need therapy.*” (Participant 10, female service user).

“*One part of it, yes, is the cost…*” (Participant 3, male service user).

“*I’m supposed to come every two weeks. If I come at the frequency of every two weeks by six, at the end of six months, that’s six by two, that’s twelve days that I would have been out of work already, which means my sick days are gone. […] My certified sick days are gone in six months.*” (Participant 12, female service user).

These contrasting forces illustrate a nuanced help-seeking landscape in which peer networks serve as critical facilitators, yet cultural norms and fears of social consequences limit emerging adults’ willingness to access formal care.

## 4 Discussion

The preliminary findings of this study highlight how stakeholders in Barbados conceptualize and perceive the health system response to the mental health needs of emerging adults. The findings illustrate the complexity of meeting the mental health needs of emerging adults in Barbados, including key facilitating and barricading factors.

### 4.1 Perspectives on mental health and mental illness

Participants described mental health as overall emotional, psychological, and social wellbeing. The emerging adults conceptualized mental health holistically. This aligns with the World Health Organization’s (WHO) definition of mental health: “Mental health is a state of mental wellbeing that enables people to cope with the stresses of life, realize their abilities, learn and work well, and contribute to their community” (46). On the other hand, participants described mental illness as a diagnosable condition like anxiety or depression. This was similarly aligned with the WHO’s definition of mental disorder, “A mental disorder is characterized by a clinically significant disturbance in an individual’s cognition, emotional regulation, or behavior. It is usually associated with distress or impairment in important areas of functioning” (47).

Emerging adults had a strong familiarity with the topic of mental health, including its definition, differences between mental health and mental illness, causes, and signs and symptoms of mental illness. However, participants noted that they cannot tell an individual has a mental illness by looking at them and that they themselves sometimes did not know they were experiencing mental health challenges. While emerging adults may adequately understand the concept of mental health, their view of mental illness is that it is complex and may be difficult to recognize. These perspectives suggest that there are potential gaps in mental health literacy and awareness that may contribute to why emerging adults may delay seeking support. This finding aligns with previous research, which suggests that health literacy and problems with symptom recognition were major barriers to help-seeking in adolescents and young adults (48). Unlike previous studies, this study highlights the importance of mental health for emerging adults as participants reaffirmed mental health as a valued asset and some identified helpful strategies to achieve good mental health. This perspective is driven by greater awareness and a focus on proactive wellness strategies.

### 4.2 Framing emerging adulthood

The findings of this study demonstrate that emerging adulthood in Barbados is outlined by both client and provider stakeholders as a uniquely challenging period characterized by expanding autonomy, heightened responsibility, and intensified pressure to achieve developmental milestones. This aligns with international research describing emerging adulthood as a period of identity exploration, instability, and heightened vulnerability (3, 49), but the Barbadian context introduces additional pressures related to financial expectations and socioeconomic precarity, and limited labor market opportunities (50). Successful management of this phase is necessary for the development of an adult identity. However, economic pressures such as the high cost of living can compound developmental stressors, heightening vulnerability to mental health difficulties. At the time of the study, youth unemployment was 22.1% over the first 6 months of 2023 and over the same period in 2024, it fell to 19.5% (50). Many emerging adults remain at home and posit that youth unemployment may create tensions in the home, as they may not be able to contribute financially.

### 4.3 Health service needs

Participants emphasized that current services do not adequately address developmental, relational, or cultural realities of emerging adults. The absence of youth-friendly environments and transitional supports undermines continuity of care. In addition. Service environments, communication styles, and care models were perceived as misaligned with emerging adults’ preferences. Participants wanted support across the lifespan, starting with curricular-based interventions in secondary schools, including inclusion of essential elements of mental health and wellness in the HFLE syllabus, as well as more guidance counsellors. There has been an ongoing call in Barbados for more mental health professionals in schools driven by increased student challenges post-pandemic, high student-to-counsellor ratios, and rising issues like violence and behavioral problems, with the Ministry of Educational Transformation actively increasing support services and professionals like psychologists, social workers, and counsellors to address these gaps and strengthen student wellbeing, though funding and implementation remain key concerns (51, 52).

### 4.4 Facilitators and barriers to help-seeking

Participants identified psychosocial support as playing a significant facilitative role. Psychosocial support refers to the actions that meet the psychological and social needs of individuals (53). For emerging adults, support from family and peers was most relevant.

Stigma continues to impede help-seeking (53). Stigma may be either ‘experiential’ targeting how stigma is experienced or ‘action-oriented’ (54). Experiential stigma includes perceived, endorsed, anticipated, received, and enacted variants whereas action-oriented stigma includes manifestations such as self-stigma, courtesy stigma, public stigma, provider-based stigma, and structural stigma (54). Of importance in this research is self-stigma which is observed in individuals with mental health conditions who internalize society’s negative stereotypes about their illnesses (54). This was in some cases potentiated by negative attitudes of relatives regarding publicly provided services. Regardless of the source or type, stigma delayed help-seeking, as has been demonstrated in the literature (48).

These insights underscore the urgent need for culturally grounded, developmentally sensitive mental health reforms that empower emerging adults and enhance their long-term resilience.

### 4.5 Strengths and limitations

A key strength of this study is its focus on an under-researched population, namely, emerging adults in a Caribbean SID, using an interpretivist, qualitative approach that centers stakeholder perspectives and lived experience. By incorporating voices from service users and service providers, the study captures a multidimensional understanding of emerging adulthood and mental health system needs in Barbados, adding contextual nuance often missing from global mental health literature. The study also benefits from rigorous methodological procedures, including reflexive journaling, peer debriefing, and iterative, rapid qualitative analysis, which support credibility and depth. While the qualitative design provided a nuanced understanding of stakeholder experiences, several limitations should be acknowledged. First, online interviews may have reduced rapport and limited the capture of nonverbal cues. Second, the perspectives of service users who do not identify as Black Barbadians, the male ‘voice’ and additional service provider views will need further exploration in the extended study. Third, research subjectivity is inherent in interpretivist research; however, reflexive journaling, field notes, contact summary sheets, and peer debriefing were used to enhance analytic trustworthiness and minimize researcher bias. Lastly, the sample size, while appropriate for qualitative inquiry, may not reflect the full diversity of experiences across all emerging adult subgroups in Barbados, hence there is scope to speak to emerging adults in other settings such as tertiary counselling centers. Despite these limitations, the study offers valuable insights that can inform targeted reforms in youth mental health policy and practice.

### 4.6 Implications of the study

The findings highlight the urgent need for mental health policies in Barbados to explicitly recognize emerging adulthood as a distinct developmental stage requiring tailored support. Health service provision should involve youth voices in the planning and evaluation stages. While current resource constraints may not allow for a bespoke clinic for emerging adults, health providers can be sensitized to adopt approaches that address the developmental needs of this group. Policies should ensure that youth-specific needs such as identity development, financial stress, role transitions, and family obligations are directly addressed. Emerging adults are calling for, among other things, life skills. This underscores the need for collaboration between the Ministry of Health and Wellness, the Ministries of Educational Transformation and Training and Tertiary Education, and the Ministry of Youth, Sports, and Community Empowerment. Similar need exists for collaboration across government ministries responsible for health, education, labor and social security; the Third Sector (non-governmental or non-profit organizations that are independent of the public and private sectors) (55); and community-based youth empowerment groups to ensure a cohesive and culturally grounded approach to mental health for emerging adults.

The vivid descriptions of the sometimes ‘invisible nature’ of mental illness highlighted that many emerging adults are entering the care pathway late due to delayed diagnosis. This is compounded by the fact that crisis presentations are the events that ultimately remove the cloak of invisibility. This, juxtaposed against the contemporary increase in suicides and other mental health disorders imply that emerging adults will benefit from routine screening for mental health conditions in the same way that individuals seeking help for physical illnesses may be screened for other noncommunicable diseases (NCDs) to avert mental health crises. For mental health service providers, the results underscore the importance of delivering care that is age-appropriate, culturally attuned, and responsive to the lived realities of emerging adults. Service delivery models should be redesigned to incorporate holistic, person-centered interventions that move beyond clinical symptom management to include psychosocial, economic, and developmental support. This may include flexible service hours for working youth, integrated psychological counselling and financial coaching in response to the stress of financial management issues reported by emerging adults in this study. Additional elements should include strengthened career-readiness programs, and family-inclusive interventions that acknowledge the fluidity of roles and responsibilities during emerging adulthood.

The findings point to several areas for future inquiry. Continued examination of post-pandemic trends in mental health service utilization by emerging adults will be critical for monitoring system capacity and anticipating future needs.

## 5 Conclusion

In this study, emerging adults in Barbados see mental health as holistic, not just the absence of disease. Additionally, participants want to see mental health embedded across the life course. However, persistent challenges such as service limitations, stigma, and poor health seeking behavior limit the ability of emerging adults to optimize their mental health and wellbeing. There is scope for a positive psychology approach to mental health and wellbeing, which is centered on the specific developmental needs of emerging adults. Positive psychology focuses on promoting positive emotions and feelings of connectedness with providers (56) and aligns with needs expressed by study participants.

## Data Availability

The raw data supporting the conclusions of this article will be made available by the authors, without undue reservation.
